# Novel AgoshRNA molecules for silencing of the CCR5 co-receptor for HIV-1 infection

**DOI:** 10.1371/journal.pone.0177935

**Published:** 2017-05-24

**Authors:** Elena Herrera-Carrillo, Ben Berkhout

**Affiliations:** Laboratory of Experimental Virology, Department of Medical Microbiology, Center for Infection and Immunity Amsterdam (CINIMA), Academic Medical Center, University of Amsterdam, Amsterdam, the Netherlands; University Hospital Zurich, SWITZERLAND

## Abstract

Allogeneic transplantation of blood stem cells from a CCR5-Δ32 homozygous donor to an HIV-infected individual, the “Berlin patient”, led to a cure. Since then there has been a search for approaches that mimic this intervention in a gene therapy setting. RNA interference (RNAi) has evolved as a powerful tool to regulate gene expression in a sequence-specific manner and can be used to inactivate the CCR5 mRNA. Short hairpin RNA (shRNA) molecules can impair CCR5 expression, but these molecules may cause unintended side effects and they will not be processed in cells that lack Dicer, such as monocytes. Dicer-independent RNAi pathways have opened opportunities for new AgoshRNA designs that rely exclusively on Ago2 for maturation. Furthermore, AgoshRNA processing yields a single active guide RNA, thus reducing off-target effects. In this study, we tested different AgoshRNA designs against CCR5. We selected AgoshRNAs that potently downregulated CCR5 expression on human T cells and peripheral blood mononuclear cells (PBMC) and that had no apparent adverse effect on T cell development as assessed in a competitive cell growth assay. CCR5 knockdown significantly protected T cells from CCR5 tropic HIV-1 infection.

## Introduction

**Fig 1:**
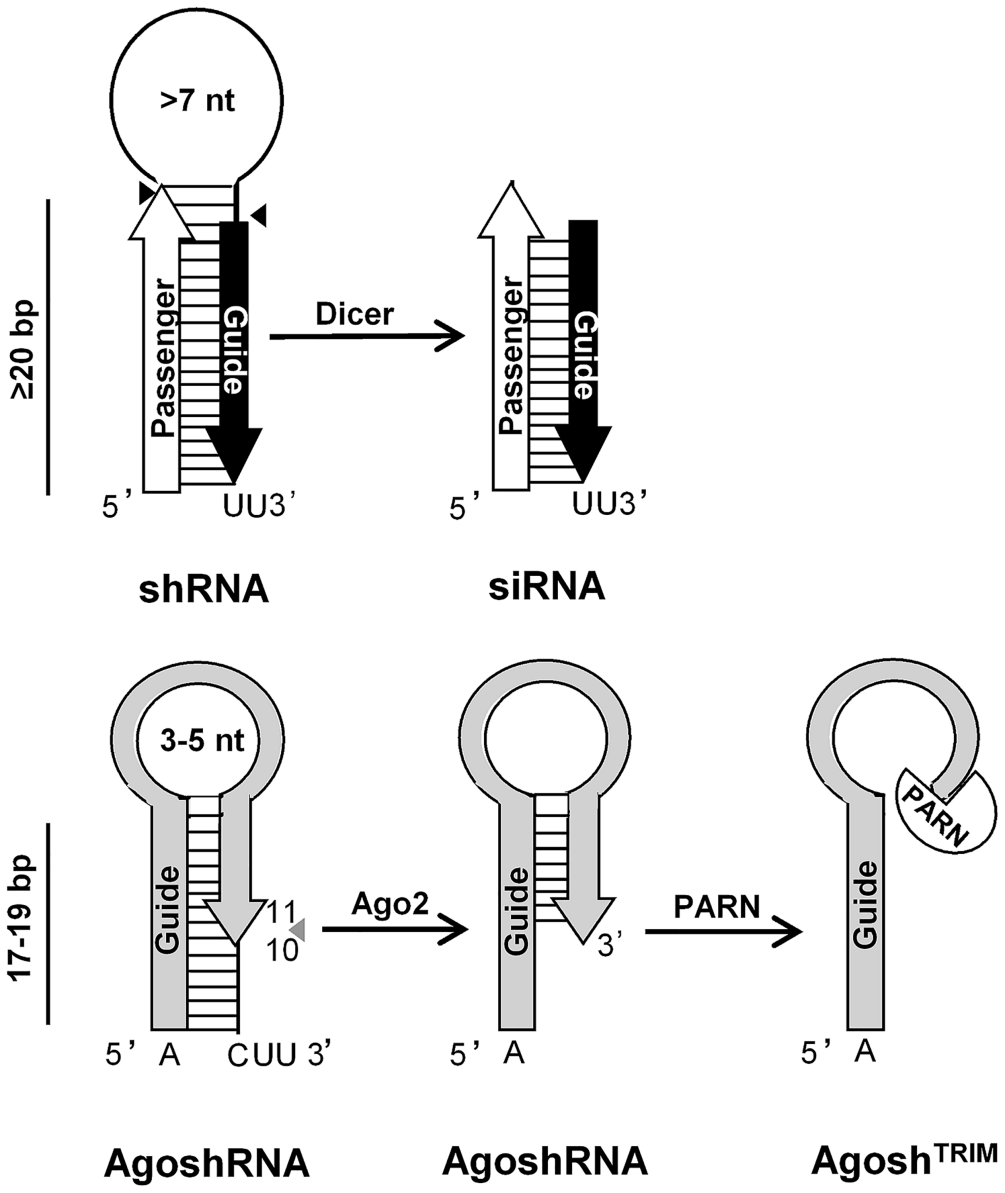
Schematic of a regular shRNA (top) and AgoshRNA molecule (bottom). In the canonical pathway the stem of the shRNA is cleaved by Dicer into an siRNA duplex of ~21 bp with a 3’ UU overhang that is loaded into RISC. One strand (the passenger, white arrow) is cleaved and degraded, the other acts as guide (black arrow) in RNAi-silencing. Alternatively, AgoshRNA is recognized directly by Ago2, triggering cleavage on the 3’ stem of the duplex between bp 10 and 11, counted from the 3’-end, yielding a single guide RNA molecule of ~30 nt (grey arrow). The predicted Dicer and Ago2 cleavage sites are marked with black and grey arrows, respectively. AgoshRNA subsequently may instruct Ago2 for RNAi-silencing or may be trimmed by PARN to create an unpaired ~24 nt guide named Agosh^TRIM^. Base pairs: bp, nucleotides: nt.

HIV-1 causes a chronic infection and no viral clearance occurs. Infected patients require long-term anti-retroviral therapy (ART) that can cause important adverse effects and allows the persistence of a latent viral reservoir [1, 2]. The effect of ART can be impaired by the selection of drug-resistant HIV-1 variants, especially when therapy adherence is sub-optimal. Gene therapy approaches have been proposed that should ideally provide a durable antiviral effect, preferentially upon a single treatment. RNAi has evolved as a powerful tool to regulate gene expression in a sequence-specific manner at the post-transcriptional level. The RNAi mechanism uses double-stranded RNA molecules (dsRNA) to trigger mRNA inactivation by cleavage. Man-made shRNAs enter the RNAi pathway halfway as Drosha processing is not needed to remove any flanking sequences. The shRNAs are processed by the cytoplasmic Dicer endonuclease that generates the small interfering RNA (siRNA) with the active guide strand and the supposedly inactive passenger strand (Fig 1, upper panel). The guide strand programs the RNAi-induced silencing complex (RISC) to cleave mRNAs with a perfect sequence complementarity. However, off-target effects via silencing of unrelated mRNAs may be caused by the passenger strand that is sometimes used by RISC instead of the guide strand [3–5].

Recently, we identified a specific shRNA design with a short stem (17–19 base pairs (bp)) and small loop (3–5 nucleotides (nt)), termed AgoshRNA, which is processed by an alternative Dicer-independent route [6–10]. AgoshRNAs are recognized by the Ago2 enzyme, which cleaves the duplex on the 3’ side between bp 10 and 11, generating a single extended ~33 nt guide strand, thus reducing the chance of off-target effects (Fig 1, lower panel). Subsequent 3’-end processing by the PARN exonuclease will generate the ~24 nt Agosh^TRIM^ molecule [9]. Another advantage of AgoshRNA inhibitors over regular shRNAs is their ability to maintain antiviral activity in Dicer-deficient monocytes that lack Dicer expression (Herrera-Carrillo et al., in preparation), which is especially important for inhibition of HIV that replicates in these cells [11, 12]. The shorter duplex of AgoshRNA inhibitors compared to regular shRNAs may further improve the safety profile because innate immunity sensors are less likely triggered [13].

We previously reviewed the ins and outs of using the RNAi machinery for a specific and durable attack on HIV-1 [14, 15]. Targeting of the viral RNA genome is prone to the selection of mutations that trigger viral escape. Targeting of cellular co-factors is restricted by the potential of adverse toxic effects on cell physiology. When protecting cells against HIV-1, it seems imperative to block the virus at an early stage, ideally even before it enters the cell or before it deposits its DNA genome—upon reverse transcription—in that of the host cell. As the major co-receptor for HIV-1 infection, the CCR5 molecule forms an ideal target for anti-HIV therapy because this protein—quite surprisingly—seems to be redundant. A proof of concept was provided by the so-called “Berlin patient”, who remained free of detectable HIV after a double stem cell transplant from a CCR5-Δ32 homozygous donor [16, 17]. However, this procedure remains risky and the logistics of finding a good donor are not easy, and this success in a single patient has in fact not yet been reproduced [18]. There has been much attention for CCR5 targeting in gene therapy approaches, e.g. targeting the gene with zinc-finger, TALEN or CRISPR-Cas nucleases [19–22]. Different RNAi screens focused on the design of siRNAs (shRNAs) against CCR5 [23–25]. However, a high shRNA expression level may be disadvantageous or even toxic to cells because of competition with the endogenous microRNA processing pathway, induction of the interferon response or off-target effects [13, 26–29]. A shRNA directed to CCR5 (sh1005) has been shown to efficiently downregulate CCR5 when introduced via a hematopoietic stem cell transplant, apparently without cytotoxicity [23, 30]. Indeed, the identification of potent RNAi reagents that are non-cytotoxic is a critical issue for therapeutic settings. In this study, we designed and tested AgoshRNA molecules for disruption of CCR5 expression.

## Materials and methods

### Plasmid construction

For the construction of sh1005 and AgoshRNA variants, complementary DNA oligonucleotides encoding the AgoshRNA sequence were annealed to create sticky BamHI and HindIII ends and subsequently inserted into corresponding restriction sites of the pSUPER vector [31]. All hairpin RNA constructs were sequence-verified using the BigDye Terminator Cycle Sequencing kit (ABI). For sequencing of hairpin RNA constructs a sample denaturation temperature of 98°C was used and 1M Betaine was included in the reaction mixture. The CCR5 coding region was PCR amplified from the pBABE-CCR5 that was obtained from the NIH AIDS Research and Reference Program (Division of AIDS, NIAID, NIH). The PCR product was digested with Xbal and cloned into the Xbal site located downstream of the luciferase gene in the pGL3 control. The orientation of the insert was checked by sequencing analysis.

### Cell culture

Human embryonic kidney 293T cells (HEK293T, ATCC CRL-11268) are adherent cells that were maintained as monolayer in Dulbecco’s modified Eagle’s medium (Life Technologies, Invitrogen, Carlsbad, CA) supplemented with 10% fetal calf serum (FCS), penicillin (100 U/ml), streptomycin (100 μg/ml) and minimal essential medium non-essential amino acids (DMEM/10% FCS) in a humidified chamber at 37°C and 5% CO_2_. PM1 and SupT1 T cells (NIH AIDS Research and Reference Program, Division of AIDS, NIAID, NIH) were grown in Advanced RPMI (Gibco BRL) supplemented with L-glutamine, 1% FCS, penicillin (30 U/ml) and streptomycin (30 μg/ml) in a humidified chamber at 37°C and 5% CO_2_. PBMC were obtained from healthy donor buffy coats (Central Laboratory Blood Bank, Amsterdam, the Netherlands) by density gradient centrifugation (ACCUSPIN System-Histopaque, Sigma Diagnostics) and frozen at high concentration in multiple vials. To minimize experimental variation between donor cell samples, we pooled the cells from four donors that are homozygous for wild-type CCR5 (CCR5wt/wt) gene in order to create a single PBMC batch for all experiments. PBMC were thawed when required and activated with phytohemagglutinin (PHA, Remel, 5 mg/ml for two days, 2 mg/ml for 3 days activation) and cultured in RPMI medium supplemented with 10% FCS, penicillin (30 U/ml), streptomycin (30 μg/ml) and recombinant interleukin-2 (rIL-2, Novartis) at 100 U/ml for 48–72 h prior to CD8+ depletion by magnetic separation (Dynal Biotech LLC, Invitrogen, Carlsbad, CA). The CD4+ cells were maintained in RPMI with 10% of FCS, penicillin (30 U/ml), streptomycin (30 μg/ml) and rIL-2 at 100 U/ml.

### CCR5 downregulation in HEK293T cells

HEK293T cells were seeded one day before transfection in 24-wells plates at a density of 1.2 x 10^5^ cells/well in 500 μl DMEM with 10% FCS, but without antibiotics. The cells were co-transfected with 100 ng of Luc-CCR5, 1 ng of pRL-CMV and increasing amounts of the AgoshRNA construct (1, 5 and 25 ng) using Lipofectamine 2000. We added pBS to create an equal DNA concentration for each transfection. Cells were lysed 48 h post-transfection to measure firefly and renilla luciferase activities using the Dual-Luciferase Reporter Assay System (Promega). The ratio between firefly and renilla luciferase activity was used for normalization of experimental variations such as differences in the transfection efficiency. An irrelevant shRNA (shNef) served as negative control, for which the luciferase activity was set at 100% expression. We performed three independent transfections, each in duplicate. Values were corrected for between-session variation as described previously [32]. The resulting six values were used to calculate the standard deviation shown as error bars.

### Lentiviral vector production and transduction

The lentiviral vector was produced and titrated as described previously [33, 34]. Lentiviral vector plasmids encoding the AgoshRNA hairpins were derived from the construct JS1 (pRRLcpptpgkgfppreSsin) [35]. The vector was produced by co-transfection of lentiviral vector plasmid and packaging plasmids pSYNGP [36], pRSV-rev and pVSV-g [37] with Lipofectamine 2000 (Invitrogen). After transfection, the medium was replaced with OptiMEM (Invitrogen). The lentiviral vector containing supernatant was collected, filtered (0.45 μm) and aliquots were stored at −80°C. The transduction titer was measured via GFP expression. PM1 T cells were transduced at an moi of 0.15 and 1.5. For selection of transduced cell clones, single GFP+ cells were selected by FACS sorting. PBMC were transduced with these lentiviral vectors at an moi of 1.5.

### Flow cytometry

To determine CCR5 receptor expression on the cell surface, PM1 T cells and PBMC were washed twice using PBS with 2% FCS and were subsequently stained with anti-CCR5 conjugated with PE-Cy7 (J418F1, Biolegend) for 30 min at room temperature. Cells were washed three times with PBS with 2% FCS and analyzed on a FACS Canto II machine. Because the percentage of GFP+ cell population differed slightly among cultures, we measured CCR5 expression as the percentage of CCR5+ cells within the GFP+ population. The data analysis was performed with Flowjo (Tree Star) software.

### HIV stocks and virus infection

Virus stocks were generated and titrated as described previously [38]. In brief, the HIV-1 BaL stock was generated by infection of PM1 T cell. The HIV-1 LAI stock was produced by transfection of HEK293T cells with the pLAI molecular clone. Cell-free viruses were passed through 0.2 μm pore-size filters and stored in aliquots at -80°C. Virus stocks were assayed to determine the tissue culture dose for 50% infectivity (TCID50) in PM1 T cell at day 14 post-infection using the classical Spearman—Kärber method. Lentivirally transduced PM1 T cells were sorted based on GFP expression. Sorted PM1 T cells (1 × 10^6^ cells in 5 ml medium) were challenged with HIV-1 BaL or LAI at low and high moi (0.01 and 0.1, respectively). Virus spread was monitored by measuring CA-p24 production twice a week for 25 days and six parallel cultures were performed per experimental condition. When HIV-1 BaL replication was apparent, cell-free virus was passaged onto SupT1 T cells to test for a potential R5-to-X4 shift in virus phenotype.

### Competitive cell growth assay

Lentivirally transduced PM1 T cells were generated using an moi of 0.15 and 1.5. Transduced PM1 T cell cultures were screened for a negative impact on cell growth by lentiviral integration and/or AgoshRNA expression using the competitive cell growth assay. In brief, the mixture of transduced (GFP+/AgoshRNA+) and untransduced (GFP-/AgoshRNA-) cells was monitored for the GFP+/- ratio over 50 days by fluorescence-activated cell sorting. The impact on cell growth was converted as percentage reduction in cell growth [39].

## Results

### Design of AgoshRNAs against CCR5 and silencing activity in transient assays

**Fig 2:**
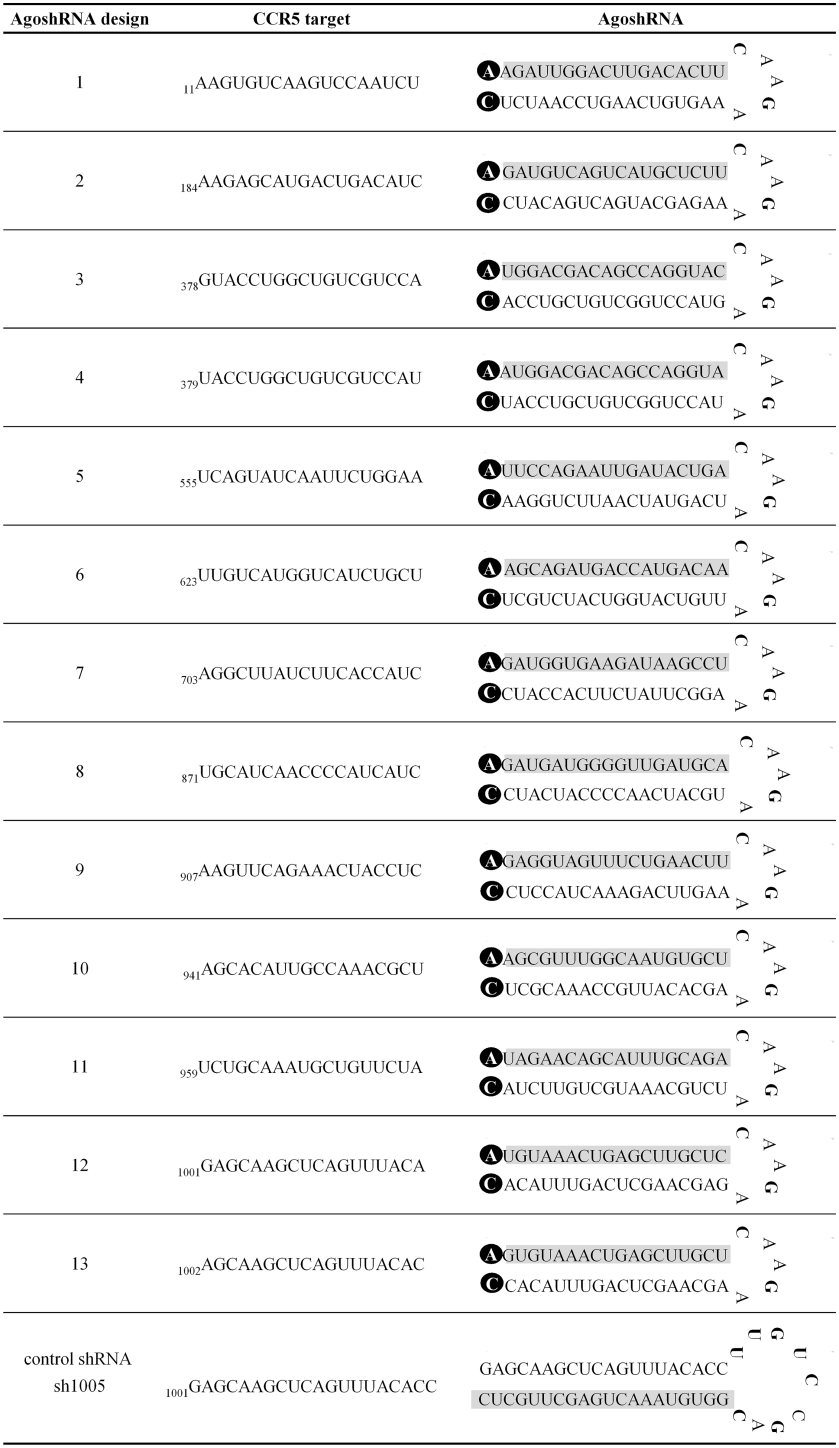
AgoshRNA molecules and CCR5 target sequences. 13 AgoshRNAs against human CCR5 were designed. The target sequences in CCR5 mRNA (GenBank: AY874120) are shown with the position in subscript. The predicted structure of the AgoshRNA molecules by MFold is shown in the third column with the guide sequence boxed in grey and the bottom mismatch A C boxed in black. The potent shRNA sh1005 was included as positive control.

We compare the regular shRNA and the novel AgoshRNA design in Fig 1, the latter yielding only a single active guide strand. Fig 2 depicts the design of 13 anti-CCR5 AgoshRNA molecules with a small 5 nt loop (CAAGA) and a duplex of 18 bp with a bottom A·C mismatch according to our most recent optimization study [40]. The guide sequence that is fully complementary to the mRNA encoding CCR5 is boxed in grey. Fig 2 also provides the actual CCR5 target sequence and position (GenBank accession number AY874120). We initially selected CCR5 target sequences using published algorithms for the design of siRNAs that lack complementarity to other cellular mRNAs (AgoshRNA 5–11) [41–48]. However, it is important to note that siRNA design algorithms may not apply to the design of shRNA and most likely also AgoshRNA molecules [49, 50]. Thus, we also decided to design AgoshRNAs that mimic previously tested shRNAs with moderate CCR5 inhibitory activity (AgoshRNA 1–4 and AgoshRNA 12–13) [24, 25]. Note that AgoshRNA targets are shorter than regular shRNA targets. Thus, two overlapping AgoshRNA molecules can be designed based on a single shRNA target sequence (AgoshRNA 3–4 and AgoshRNA 12–13). As positive control we included the effective sh1005 against CCR5 that was selected from a large screen [51].

**Fig 3:**
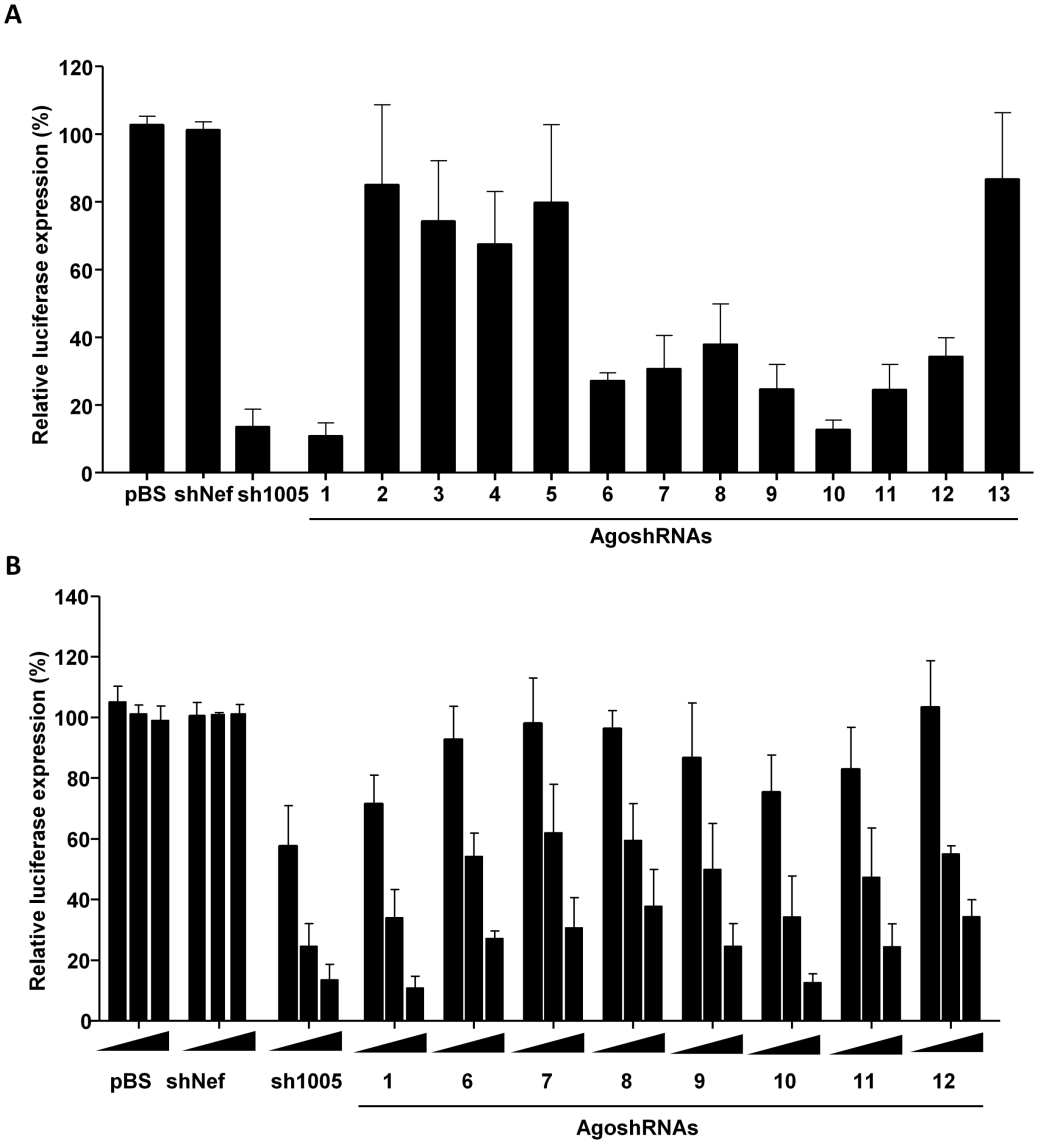
Knockdown activity of the AgoshRNAs against luciferase-CCR5. **(A)** Luciferase knockdown by the AgoshRNA was determined by co-transfection with the AgoshRNA constructs. HEK293T cells were co-transfected with 100 ng of the firefly luciferase reporter plasmid, 1 ng of renilla luciferase plasmid, and 25 ng of the corresponding AgoshRNA constructs. pBluescript (pBS) plasmid and an unrelated shRNA (shNef) served as negative control. The luciferase activity scored with shNef activity was set at 100%. **(B)** Luciferase knockdown by the most potent AgoshRNA constructs is dose-dependent when HEK293T cells were co-transfected with firefly luciferase reporter plasmid, renilla luciferase plasmid and increasing amount of AgoshRNA constructs (1, 5 and 25 ng). The mean values and standard deviation are based on six independent transfections.

The AgoshRNA molecules were expressed from plasmids with the H1 polymerase III promoter. We tested for anti-CCR5 activity in co-transfections with a luciferase reporter construct with the nearly complete CCR5 mRNA sequence inserted upstream of the poly(A) signal. Each AgoshRNA construct (25 ng) was co-transfected with the luciferase reporter (100 ng) in HEK293T cells to score for the ability to silence CCR5 expression. A fixed amount of renilla luciferase plasmid was used to control for the transfection efficiency. Two days post-transfection, the relative luciferase expression was measured (Fig 3A). The ratio between the luciferase and renilla activity obtained with 25 ng of the shNef control plasmid was set at 100%.

Eight AgoshRNAs (1, 6–12) showed moderate silencing activity on the Luc-reporter, with luciferase levels dropping below 50% of the uninhibited value. The positive control sh1005 exhibited better silencing activity than the two overlapping AgoshRNAs (12 and 13) that were based on this shRNA. Agosh12 showed modest activity, whereas Agosh13 demonstrated no activity, even though they differ by a single nt. Note that these AgoshRNAs have an 18 nt target sequences, whereas sh1005 targets a 20 nt sequence. Only two AgoshRNA designs (1 and 10) seem comparable in silencing activity to sh1005 with luciferase levels dropping to <20%. Silencing activity of the best eight AgoshRNA candidates (1, 6–12) was subsequently tested in a titration with increasing amount of the AgoshRNA constructs (1, 5 and 25 ng) (Fig 3B). The results confirm the inhibitory potency of this subset of AgoshRNA molecules, which act in a dose-dependent manner.

### Downregulation of CCR5 expression in PM1 T cells

**Fig 4:**
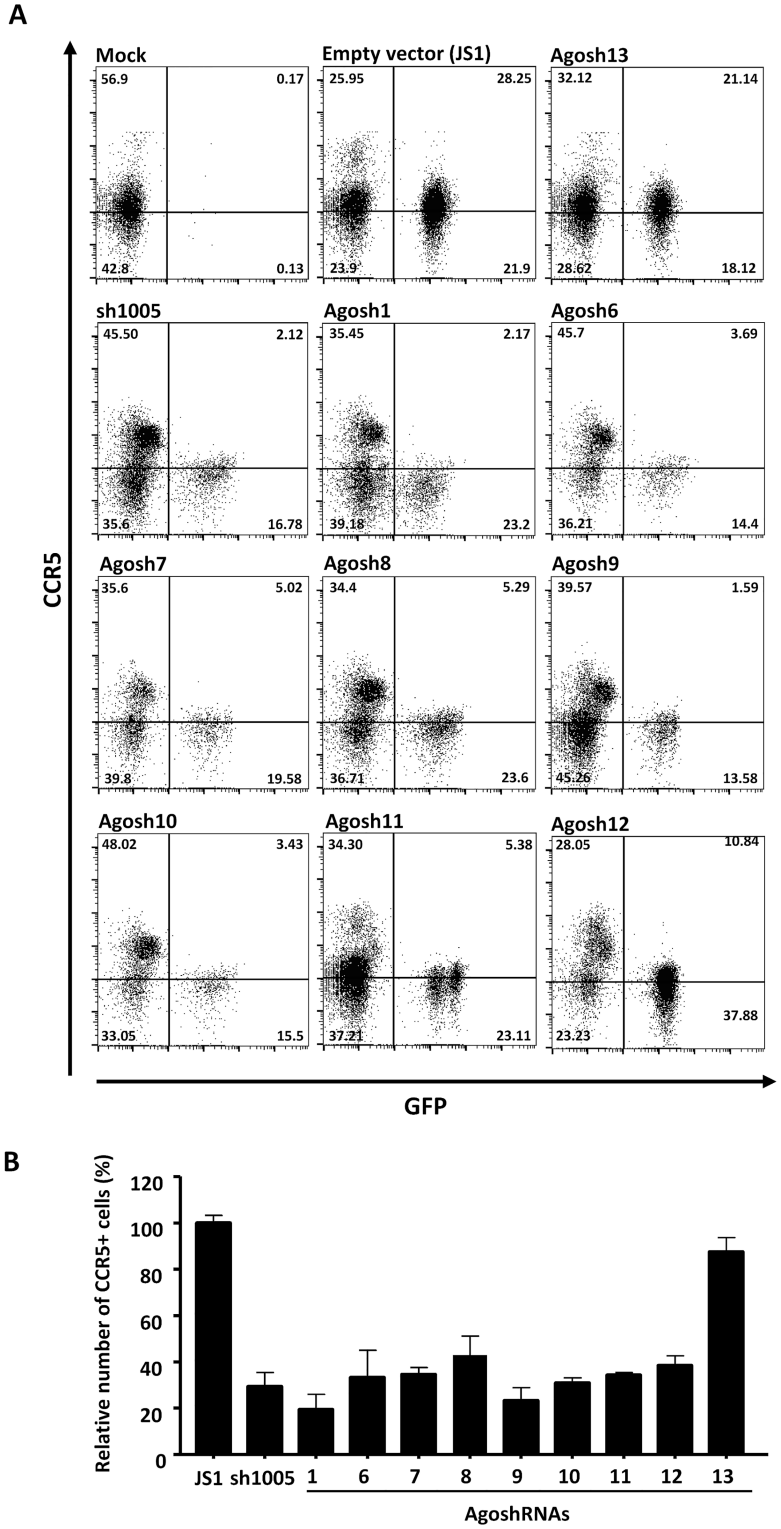
Identification of potent AgoshRNAs against CCR5. **(A)** PM1 T cells were transduced with lentiviral vectors expressing AgoshRNAs against CCR5, cultured for four days and analyzed by flow cytometry for CCR5 expression in GFP-expressing cells. **(B)** Percentage of CCR5+ cells in the GFP+ versus GFP- population in transduced PM1 T cells at day 4. The mean values and standard deviation are based on three independent experiments.

We next examined downregulation of CCR5 protein expression on the surface of PM1 T cells that were stably transduced with the AgoshRNA constructs. To achieve durable expression of the antivirals, we choose the lentiviral system (JS1 vector) that stably integrates into the cellular genome. PM1 T cells express both the CXCR4 (X4) and CCR5 (R5) co-receptors and support the replication of X4- and R5-tropic HIV-1 strains. The active subset of AgoshRNAs (1, 6–12) and a non-active AgoshRNA (13) were expressed in PM1 cells upon lentiviral transduction. We used a low multiplicity of infection (moi: 0,15) to obtain maximally a single integrated lentiviral vector per cell to avoid AgoshRNA overexpression and putative saturation of the RNAi machinery. Green fluorescent protein (GFP) is encoded by this vector and the expression of CCR5 on GFP+ cells was quantified by fluorescence-activated cell sorting (FACS) at day four post-transduction (Fig 4A). Mock-transduced cells expressed no GFP and ~50% of the cells were CCR5+. Cultures transduced with an empty vector (JS1) or the inactive AgoshRNA (Agosh13) contained ~50% GFP+ cells that showed unaltered CCR5 expression. The active AgoshRNAs (1, 6–12) selectively reduced CCR5 cell surface expression in the GFP+ population relative to the GFP- population. We plotted the percentage of CCR5+ cells in the GFP+ versus GFP- population based on three independent experiments (Fig 4B). Two AgoshRNAs (1 and 9) consistently caused profound CCR5 downregulation to less than 20% CCR5+ cells, a level of inhibition that is even stronger than that caused by sh1005. The other AgoshRNAs (6–8 and 10–12) resulted in cultures with ~40% CCR5+ cells. These results globally confirm the inhibition results obtained in transient transfections, except for AgoshRNA 10 that was more active in the luciferase assay than in HIV-1 inhibition. The target sequence may be masked by stable structures in the HIV-1 genome that are not folded in the subgenomic luciferase mRNA [52–54]. Thus, we identified AgoshRNA 1 and 9 as the most potent CCR5 inhibitors.

### Durable AgoshRNA expression and CCR5 downregulation

**Fig 5:**
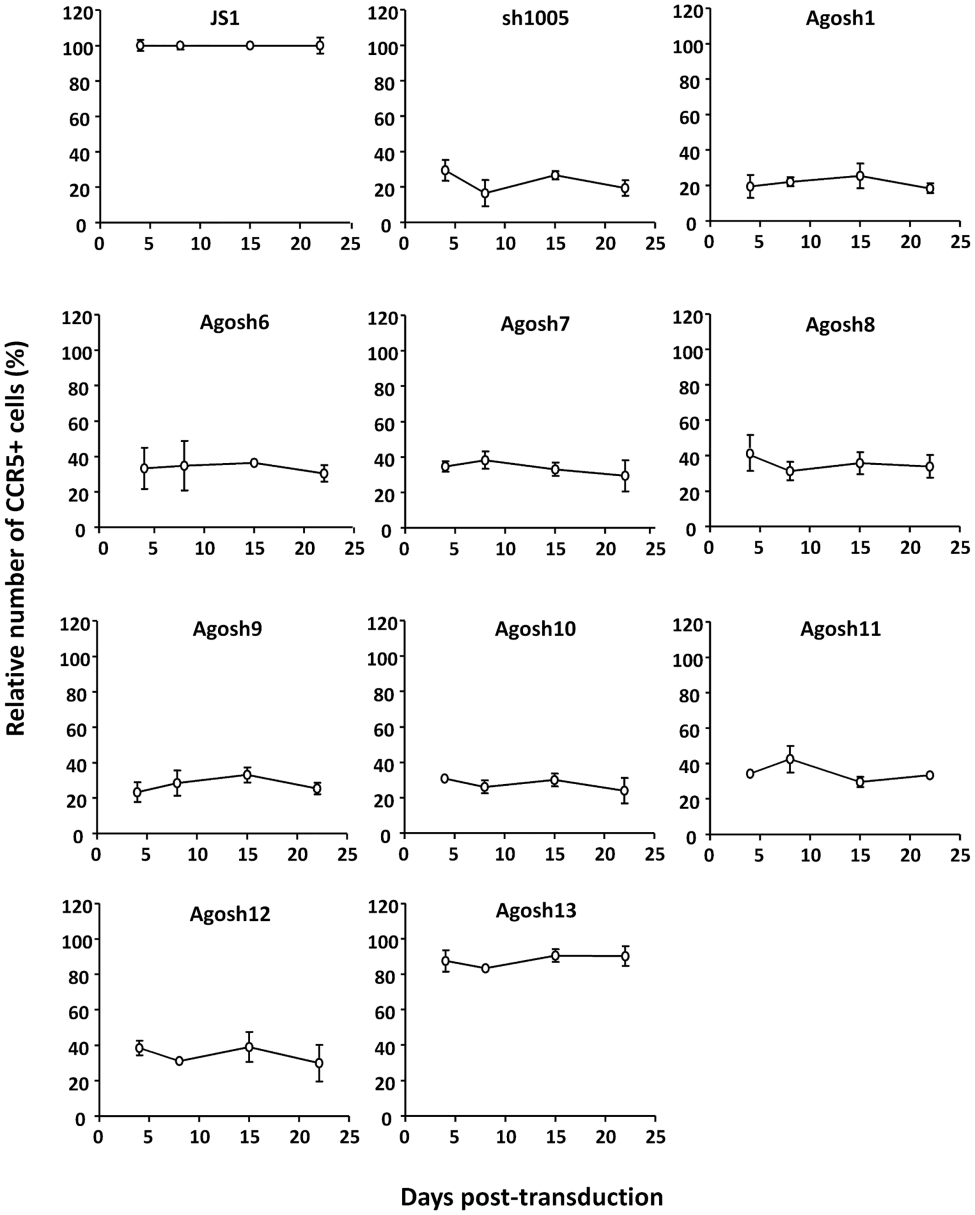
Stable AgoshRNA-mediated CCR5 silencing in PM1 T cells. PM1 T cells were transduced with lentiviral vectors expressing AgoshRNAs against CCR5, cultured for 22 days and analyzed by flow cytometry once a week for CCR5 expression in GFP-expressing cells.

To evaluate the durability of AgoshRNA-mediated CCR5 inhibition we followed these lentivirus-transduced PM1 T cells over time. We compared the most active AgoshRNAs (1, 6–12) with the inactive AgoshRNA (13) and the empty JS1 vector as negative controls. CCR5 expression was quantified by FACS starting at day four post-transduction up to day 22. We plotted the percentage of CCR5+ cells in the GFP+ versus GFP- population based on three independent experiments (Fig 5). Over time the reduced percentage of CCR5+ cells remained constant for most AgoshRNA-expressing cultures, indicating stable AgoshRNA expression and CCR5 suppression.

### Monitoring the effect of AgoshRNA expression on T cell viability

We next determined if the expressed AgoshRNAs have an adverse effect on cell viability by means of the ultra-sensitive competitive cell growth assay (CCG) [39]. We again transduced PM1 T cells at an moi of 0,15 with the lentiviral constructs expressing the subset of most active AgoshRNAs. Transduction was also performed at a high moi of 1.5 to induce AgoshRNA overexpression. Transduced GFP+ cells express the AgoshRNA and untransduced GFP- cells form the internal control. To detect any negative effect of AgoshRNA expression on cell growth, the GFP+/GFP- ratio was simply monitored for 50 days upon passage of the transduced culture (Table 1). The empty lentiviral vector (JS1) served as negative control. We included shGag5 as a toxic, positive control [14], which indeed significantly reduced the percentage of GFP+ cells over the 50 day time course. None of the AgoshRNA-transduced cultures showed such a profound impairment of cell growth, although a slight reduction of the percentage of GFP+ cells was measured for AgoshRNAs 11 and the control sh1005, but mostly at the high moi.

**Table 1:** Competitive cell growth (CCG) assay.

Lentiviral construct	Change in % GFP+ cells^a^^,^^b^	
	moi 0.15	moi 1.5
JS1	0.0	2.1
shGag5	-16.2	-25.8
sh1005	-2.3	-6.2
Agosh1	0.2	-0.5
Agosh6	-0.7	2.3
Agosh7	1.1	-0.2
Agosh8	-0.5	2.8
Agosh9	-1.2	-4.9
Agosh10	-0.2	6.3
Agosh11	-3.2	-8.7
Agosh12	5.1	-1.6

^a)^ GFP expression was measured after 50 days

^b)^ ±5% reflects experimental variation

### Downregulation of CCR5 expression in activated PBMC

**Fig 6:**
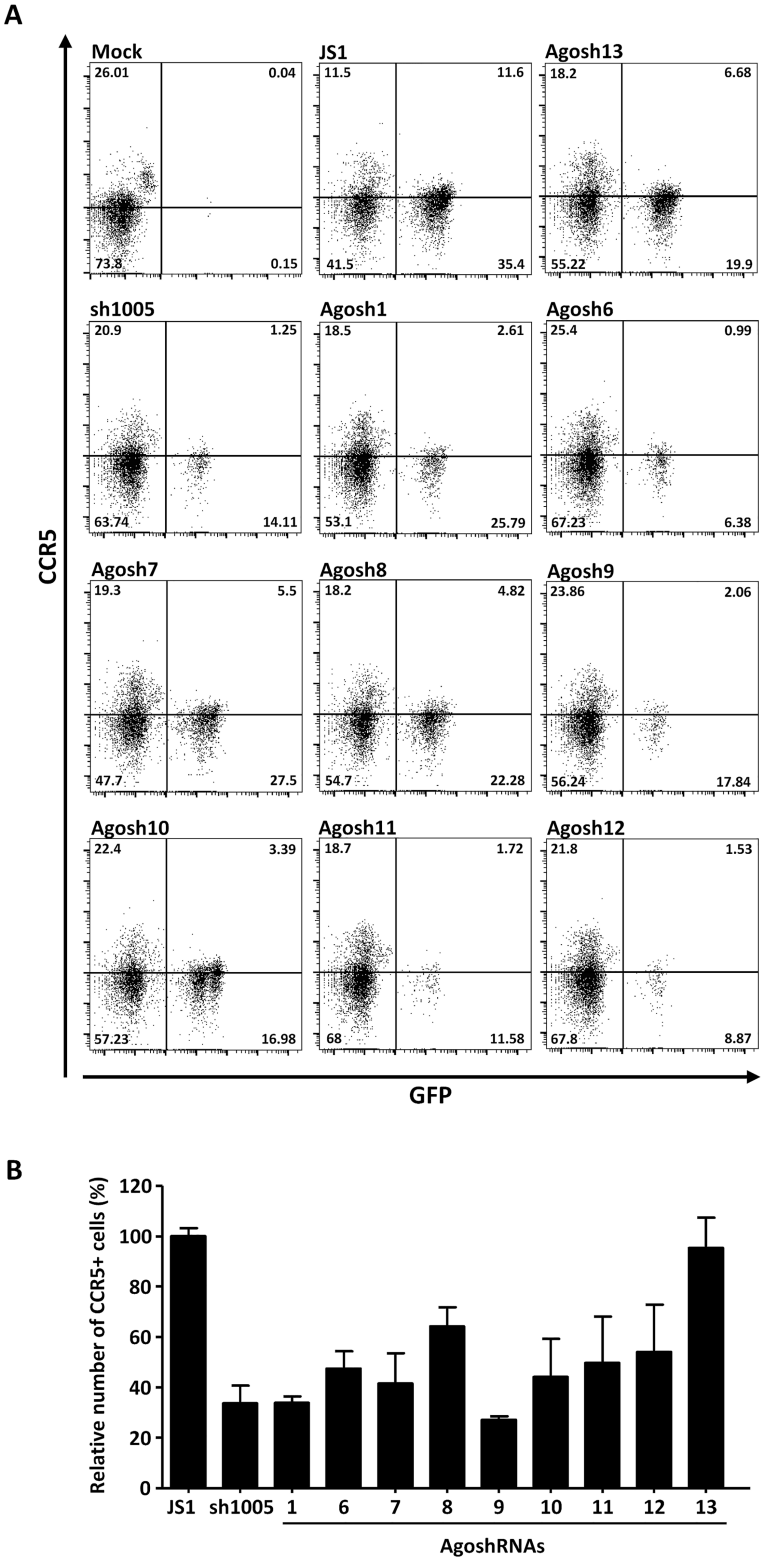
Reduction of CCR5 surface expression on human PBMC transduced by anti-CCR5 AgoshRNA lentiviral vectors. PHA and IL2-stimulated PBMC were transduced with the indicated lentiviral vector. The transduced cells were cultured in IL2-containing medium for 7 days before FACS analysis for CCR5 expression on the cell surface. **(A)** Representative FACS analyses are shown. **(B)** Percentage of CCR5+ cells in the GFP+ versus GFP- population was calculated. Three independent experiments were performed. The mean values and standard deviation are indicated.

Using a similar approach, we studied downregulation of CCR5 on the surface of PBMC at day seven post-transduction with the lentiviral vectors. The mock transduced PBMC culture contained ~26% CCR5+ cells (Fig 6A). We reached 50% transduction efficiency with JS1 in these primary cells and generally lower efficiencies were scored for the AgoshRNA vectors. JS1 transduced control cultures also scored ~25% CCR5+ cells. We observed modest CCR5 down-regulation for the AgoshRNAs. Based on three independent experiments, we calculated the relative number of CCR5+ cells in the GFP+ versus GFP- population at day seven post-transduction (Fig 6B). The results were comparable to that observed in PM1 T cells, but the magnitude of CCR5 downregulation was slightly lower in PBMC. AgoshRNA 1 and 9 again demonstrated the best result, yielding less than 40% CCR5+ cells. AgoshRNAs 6, 7, 10–12 showed moderate activity (~60% CCR5+ cells) and AgoshRNA 8 caused a minor reduction (70% CCR5+ cells).

### HIV-1 inhibition by CCR5 AgoshRNAs

**Fig 7:**
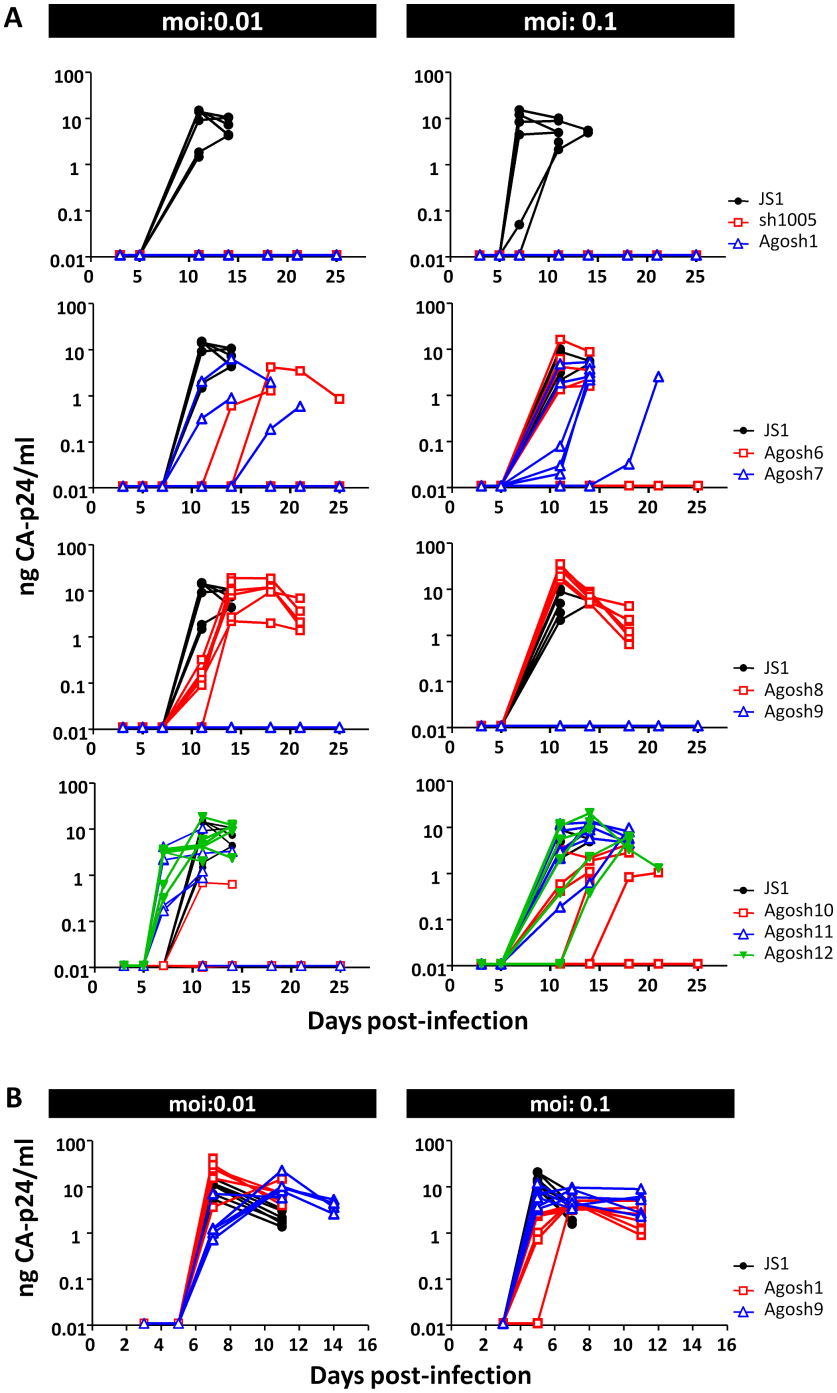
The impact of anti-CCR5 AgoshRNAs in a spreading HIV-1 infection. Stably transduced PM1 T cells expressing the AgoshRNAs variants or the sh1005 control were challenged with **(A)** the R5-tropic BaL isolate or **(B)** the X4-tropic LAI isolate at different moi: 0.01 (left panel) and 0.1 (right panel). Cells transduced with the empty lentiviral vector JS1 served as control. Virus replication was monitored by measuring CA-p24 in the supernatant for 25 days.

To test HIV-1 inhibition in a spreading virus infection, we used the PM1 T cells that were previously transduced with the lentiviral constructs encoding the best eight AgoshRNA inhibitors (1, 6–12) at an moi of 0.15. Cells were FACS-sorted for GFP expression after four days and subsequently challenged with two amounts of the R5-tropic HIV-1 isolate BaL (moi 0.01 and 0.1). Infections were performed in 6-fold because we also wanted to monitor virus evolution, which is a chance process. Cells transduced with the empty JS1 vector (and GFP-sorted) served as negative control, cells expressing sh1005 served as positive control. Viral CA-p24 production was monitored starting at day three post-infection up to day 25 (Fig 7A). All 120 infections were performed in parallel, but are plotted in eight graphs for clarity. The JS1 negative control is shown in each graph.

We will first discuss the results obtained in the low moi challenge (Fig 7A, left panels), which demonstrated exponential viral spread around day 11 in the control JS1 cultures. The sh1005 inhibitor was able to block HIV-1 spread in all six parallel cultures up to at least 25 days, when the experiment was terminated. HIV-1 replication was also blocked in all six AgoshRNA 1 and 9 cultures, but more variable results were scored for AgoshRNAs 6, 7, 10 and 11. We think that these results indicate sub-optimal virus inhibition and not the selection of X4-using escape viruses because no virus could be passaged on X4-expressing SupT1 cells (results not shown). No appreciable virus inhibition was apparent for AgoshRNA 8 and 12 (all cultures) and 11 (most cultures). At high moi (Fig 7A, right panels), sh1005 and AgoshRNAs 1 and 9 maintained strong inhibition of virus replication in all six parallel cultures, while the majority of AgoshRNA 6–8 and 10–12 cultures were permissive for HIV-1 BaL replication.

We performed an additional specificity test for the best inhibitors AgoshRNA 1 and 9. These cultures were also infected with the X4-tropic HIV-1 isolate LAI that should be insensitive to CCR5 suppression. HIV-1 LAI replication on the BaL-restricted PM1 cells was indeed not affected (Fig 7B), confirming that BaL-inhibition is due to CCR5 silencing and not caused by other LV or AgoshRNA induced effects, including non-desirable off-target effects. Taken together, we conclude that the AgoshRNA design can trigger reduced CCR5 expression that provides selective resistance against R5-tropic HIV-1 infection.

## Discussion

Gene-based therapy presents an attractive way for controlling HIV infection by a single treatment. Interest in targeting the primary CCR5 receptor increased after the remarkable success in curing HIV in the so-called “Berlin patient” after a double bone marrow transplant from a CCR5-Δ32 homozygous donor. We utilized the novel AgoshRNA design to target the CCR5 mRNA for sequence-specific silencing. AgoshRNAs differ from regular shRNAs in having a smaller loop (5 nt) and shorter stem length (18 bp). AgoshRNAs are too small to be processed by Dicer and are processed instead by Ago2 to generate a single guide strand, thus avoiding off-target effects by the passenger strand of regular shRNAs [10, 11]. In this study, we designed 13 AgoshRNAs targeting different parts of the CCR5 mRNA. Eight AgoshRNAs induced profound and dose-dependent downregulation of CCR5 expression in transiently transfected HEK293T cells and subsequently in stable lentivector-transduced PM1 T cells and PBMC. Downregulation of CCR5 from the cell surface was monitored by flow cytometry and we observed that a constant low CCR5 level was maintained over time, indicating stable AgoshRNA expression from the H1-driven transgene. No adverse cell effects or toxicity were apparent for the AgoshRNA candidates in a competitive cell growth assay. These combined findings illustrate the antiviral potency and reduced off-targeting potential of AgoshRNAs, which is an important property for the future development of small therapeutic RNAs. Nevertheless, AgoshRNA candidates should always be checked for possible adverse effects in appropriate *in vitro* and *in vivo* systems.

We tested the HIV susceptibility of PM1 T cells upon CCR5-downregulation. AgoshRNA 1 and 9 proved to be the best HIV-1 inhibitors when the modified cells were challenged with a R5-tropic virus variant. These results demonstrated that downregulation of CCR5 by AgoshRNAs is sufficient to inhibit R5-tropic HIV-1 infection. Virus inhibition was observed by a ~5-fold reduction of the number of R5-positive cells. However, the cultures remained susceptible to an X4-tropic HIV-1 variant. AgoshRNAs 6, 7, 10 and 11 showed more variable results. Even though replication of R5-tropic HIV-1 was delayed, the majority of cultures eventually became HIV-1 positive. We performed a phenotypic test on X4-expressing SupT1 T cells to characterize the tropism of these HIV-1 variants and detected no replication of X4-using variants. In other words, we likely observed break-through replication of the wild-type virus in the presence of a moderate inhibitor.

In theory, these antiviral AgoshRNAs should remain active in Dicer-deficient monocytes that play a role in HIV-1 pathogenesis and may contribute to the long-term stable HIV-1 reservoir in treated patients [55]. We previously reported that AgoshRNA molecules are processed by Ago2 and thus independent of Dicer [8, 10]. It would be scientifically interesting to test whether AgoshRNAs provide superior protection against R5-tropic strains in monocytes versus the “classical” shRNA design. However, there are several issues that complicate such an analysis. First and most importantly, the difficulty of keeping monocytes undifferentiated during this lengthy experiment. Alternatively, monocyte-derived cell lines such as THP-1 could be used, which maintain monocyte characteristics and do not express Dicer, but these cells cannot be infected by R5-tropic strains [56–58]. Differentiation into mature macrophage-like CCR5-positive cells could be induced by phorbol 12-myristate 13-acetate (PMA) [59], but these cells will also start expressing Dicer, which is incompatible with our experimental strategy.

It is important to note that the vast majority of patients harbor R5-tropic strains, which are also most commonly transmitted. R5-tropic variants are predominant during early stages of infection, while X4-tropic strains can emerge later in some patients, usually concomitant with disease progression [60–62]. Individuals with a homozygous CCR5-d32 deletion do not express any CCR5 co-receptor and are highly protected from HIV-1 acquisition [63]. A proof of therapeutic concept is provided by the “Berlin patient” who has stayed free from any viral rebound for a period of eight years [64]. Not any replication competent virus could be detected when drug therapy was stopped, indicating that the patient was functionally cured. However, viral escape by emergence of X4-tropic viruses may occur as observed in the “Essen patient” case. This patient received a treatment similar to that of the “Berlin patient”, but the viral load of the patient rebounded during engraftment, yielding an HIV quasi-species that was able to use the CXCR4 co-receptor [65].

To improve antiviral efficacy and to address potential viral escape routes, one has to consider combinatorial strategies [66–69]. One possibility is dual entry inhibition by targeting both the CCR5 and CXCR4 co-receptors. Nevertheless, the impact of CXCR4 disruption in humans remains a major concern because this co-receptor is widely expressed on the surface of many cell types and involved in multiple physiological processes [70]. Another possibility is to combine inhibitory agents that act a different virus entry steps. For instance, the C46 peptide is expressed as a transmembrane protein and effectively inhibits fusion of the viral and cellular membranes during virus entry [71]. Another combinatorial option is the inclusion of AgoshRNAs that target HIV-1 RNA.

In sum, we demonstrated that potent AgoshRNAs can be generated against the CCR5 co-receptor of HIV-1. We verified the absence of cytotoxic effects and demonstrated that a sustained antiviral effect is caused by these AgoshRNAs. These results suggest that the future for AgoshRNA therapeutics is promising.
